# Penicillin-allergy delabelling resources for clinicians practicing in resource-limited settings: a full educational resource review of the grey literature

**DOI:** 10.1093/jacamr/dlad014

**Published:** 2023-03-20

**Authors:** Mary L Staicu, Meghan N Jeffres, Bruce M Jones, Kayla R Stover, Jamie L Wagner, Christopher M Bland

**Affiliations:** Department of Pharmacy, Rochester General Hospital, Rochester, NY, USA; Department of Clinical Pharmacy, University of Colorado Skaggs School of Pharmacy and Pharmaceutical Sciences, Aurora, CO, USA; Department of Pharmacy, St. Joseph’s/Candler Health System, Inc., Savannah, GA, USA; Department of Pharmacy Practice, University of Mississippi School of Pharmacy, Jackson, MS, USA; Department of Pharmacy Practice, University of Mississippi School of Pharmacy, Jackson, MS, USA; Department of Clinical and Administrative Pharmacy, University of Georgia College of Pharmacy, Savannah, GA, USA

## Abstract

**Background:**

The clinical and financial consequences associated with a penicillin-allergy label are increasingly evident and have garnered support from international organizations to prioritize penicillin-allergy delabelling programmes. Most settings lack access to resources including drug allergy specialists and rely on general practitioners (GPs) and pharmacists.

**Objectives:**

The aim of this scoping review was to identify and describe freely available penicillin-allergy delabelling materials to guide clinicians practising in resource-limited settings with initiative application.

**Methods:**

This scoping review searched two grey literature databases, six targeted websites and consulted content experts to identify freely available materials in the English language that provided evidence-based and actionable penicillin-allergy delabelling strategies. Study investigators ranked and voted on which screened resources should be included in the final review. Characteristics of resources were evaluated and compared.

**Results:**

Out of 1191 total citations, 6 open-access resources were included. Penicillin-allergy toolkits featuring various delabelling strategies were identified in four resources. The toolkits supported a broad range of downloadable and adaptable materials, predominantly targeted towards GPs. Patient educational materials were also provided. Another resource highlighted a point-of-care penicillin-allergy risk assessment calculator via a free mobile app that quickly and accurately identified low-risk penicillin-allergic patients. The final resource, a supplemental instructional video, presented impactful and standardized delabelling strategies that clinicians can adopt into daily practices.

**Conclusions:**

Limited penicillin-allergy delabelling materials are available in the grey literature but existing resources provide broad and diverse opportunities. Additional support from health protection agencies is critical to augment ongoing delabelling efforts.

## Introduction

The consequences of the penicillin-allergy label have become increasingly evident over the past few decades. From higher rates of multidrug-resistant infections, such as MRSA and VRE, to increased exposure to high-risk healthcare settings including longer hospital admissions and more frequent outpatient appointments, the public health implications alone have stimulated support for penicillin-allergy delabelling programmes from established organizations.^1–5^ Early data show that the removal of erroneous penicillin-allergy labels collectively improves patient and healthcare-related outcomes.^3^

Conservative estimates indicate that up to 10% of the world’s population are labelled as having an allergy to penicillin.^6^ The vast majority of these potential 800 million individuals, however, will prove tolerant to penicillin if rechallenged, and bear the risks of this inaccurate label unnecessarily.^7,8^ The implications of penicillin-allergy mislabelling and its cumulative sequelae have yet to be fully appreciated on a global scale, especially in low-income (LICs), lower-middle income (LMICs) and high- and middle-income countries (HMICs).^9^

Despite the extent of the challenge at hand, access to allergy specialists remains largely insufficient in many parts of the world, with growing allergist shortages reported by national and international organizations. Even in countries with allergy and immunology training programmes for physicians, such as the USA, the ratio of penicillin-allergic patients per board-certified allergist exceeds 5000, with more than 50% of hospitals lacking inpatient consultative services and up to 80% of counties lacking practising allergists altogether.^10–13^ This is a particular problem in LICs, LMICs and UMICs, where the study of allergy in medicine is not yet considered a specialty, leaving most providers without sufficient training in basic allergy management.^9^ Further, outpatient appointment waiting times for a penicillin-allergy assessment range from 60 days to up to 7 years, as reported in some regions of the world.^14,15^

Given such deficits, readily available and actionable strategies that empower non-allergist clinicians are desperately needed to supplement existing delabelling efforts. This scoping review aims to identify and characterize freely accessible, grey-literature materials to guide non-allergy clinicians, especially those practising in resource-limited settings, on penicillin-allergy delabelling initiatives.

## Methods

This scoping review followed the framework outlined by Arksey and O’Malley, consisting of six stages designed to strategically answer the research question.^16,17^

### Stage 1. Identifying the research question

This review sought to assess penicillin-allergy delabelling materials in resource-limited settings, including LMICs. It was designed to answer the following research question: ‘What grey-literature materials are available to guide clinicians practising in resource-limited settings with penicillin-allergy delabelling initiatives?’ The following objectives were proposed:

1. Identify grey-literature resources that assist with penicillin-allergy delabelling practices.
2. Select penicillin-allergy resources to enhance delabelling within an individual clinical practice.
3. Analyse and compare characteristics of various penicillin-allergy delabelling resources.
4. Evaluate opportunities to enhance penicillin-allergy delabelling strategies.

### Stage 2. Identifying resources

A grey-literature search was conducted using a three-pronged approach to identifying penicillin-allergy delabelling resources:

1. Grey literature databases (WorldCat; Agency for Healthcare Research and Quality).
2. Targeted websites [American Academy of Allergy, Asthma, and Immunology (AAAAI); American College of Allergy, Asthma, and Immunology; WHO; IDSA; YouTube; Twitter].
3. Study investigator nominations.

The following terms were used to search grey-literature databases as both keywords in the title and/or subject headings, as appropriate: (‘penicillin allergy’ OR ‘penicillin hypersensitivity’) AND (‘delabel*’ OR ‘remov*’ OR ‘assess*’). For other databases, including targeted websites, less restrictive search terms were used.

### Stage 3. Selection of resources for review

A two-step method was applied for resource selection. In the first step, two researchers (M.L.S. and M.N.J.) independently screened titles and content of citations returned from the grey-literature search. In the second step, the two investigators identified the final list of resources based on applied inclusion and exclusion criteria. Resources were included if they were published in the English language, were available as open access, and if their resource content addressed the research question by providing actionable penicillin-allergy delabelling strategies. Resources were excluded if they were published in an academic journal, book or other traditional peer-reviewed publication channels. The final list of resources was distributed to study investigators via a web-based survey, who then ranked and voted on which resources should be included in the scoping review. A 5-point Likert scale (1 = strongly disagree, 2 = disagree, 3 = neither agree nor disagree, 4 = agree, 5 = strongly agree) was utilized to assess the study investigator’s level of agreement with the following statements:

1. The resource content is accurate and in accordance with national guidelines and practices.^7^
2. The resource content provides actionable and impactful penicillin-allergy delabelling strategies.

Discrepancies in resource selection were reviewed and a consensus was reached within the team. Top scoring resources were selected for final review and data extraction.

### Stage 4. Data extraction

Characteristics of included resources were determined using a standardized data extraction form. The following information was evaluated: resource type, source of resource, focus of topic, target audience, relevance and setting of resource.

### Stage 5. Data summary and synthesis of results

Extracted data were mapped based on individual resource characteristics, prioritizing results based on relevance to the research question. Opportunities for future efforts in line with the study objectives were also identified.

### Stage 6. Consultation

To provide insights into the process, local stakeholders involved with penicillin-allergy education and delabelling practices were invited to participate in the process.

## Results

**Figure 1.**
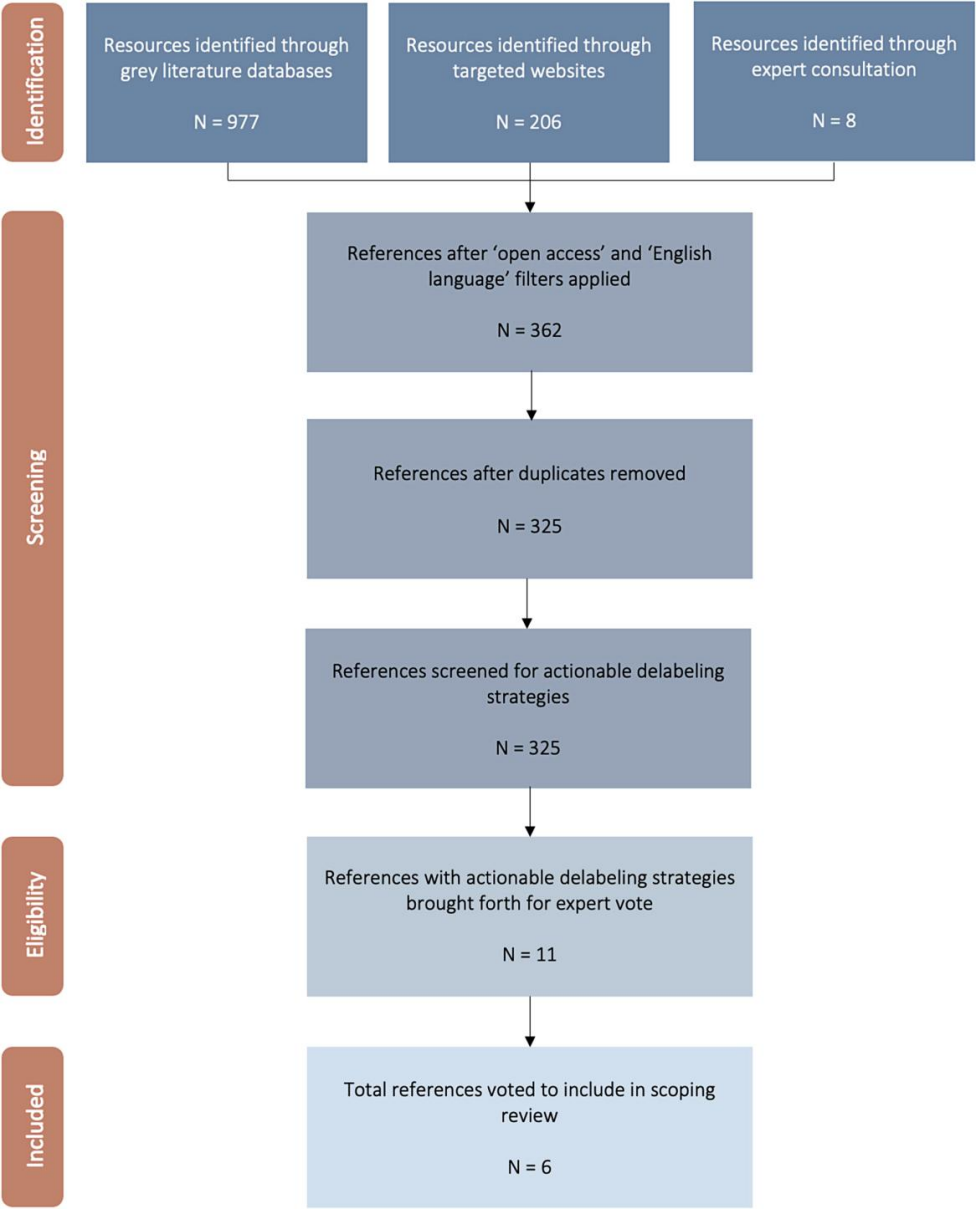
Flow diagram of scoping review process and reference identification.

**Figure 2.**
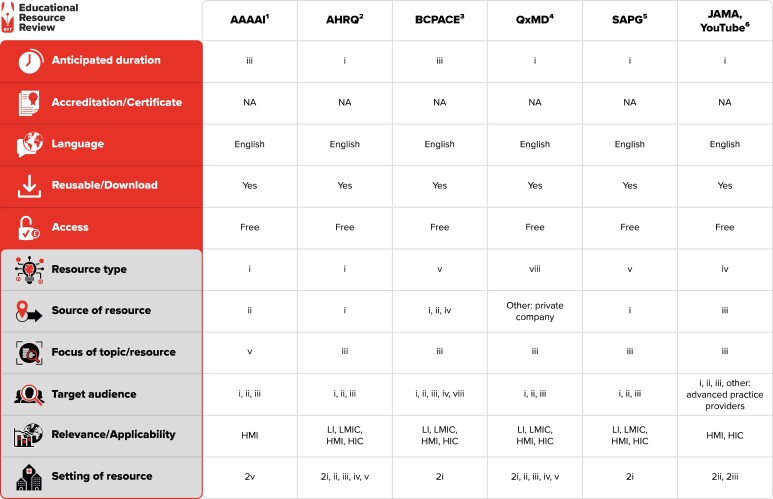
General characteristics of resources included in the scoping review. Anticipated duration: (i) short <4 h; (ii) long >4 h; (iii) self-paced. Resource type: (i) website online reading material and other resource; (ii) website primarily aimed at news items; (iii) online/distance learning courses [massive open online course (MOOC), unfacilitated courses, online modules]/community of practice; (iv) webinars, video, online lectures, podcasts, animation video, maps, photos; (v) clinical practice antimicrobial stewardship materials: PDFs, PowerPoints, newsletters, infographics, pamphlets, e-portfolios, workbooks; (vi) guidelines, policies, handbooks; (vii) material from face-to-face non-e-learning courses (programmes, teaching materials etc. from workshops, lectures, seminars); (viii) e-books via apps; (ix) public, media, political awareness and engagement materials; (x) commercial advertising (TV, radio, film, social media); (xi) evidence: by systematic reviews/meta-analysis in relation to antimicrobial resistance; (xii) datasets, compelling/illustrative case studies on antimicrobial stewardship; (xiii) patient stories. Source of resource: (i) governments; (ii) professional societies; (iii) universities/higher education institutes; (iv) healthcare facilities; (v) WHO; (vi) industry; (vii) insurance companies; (viii) non-governmental organizations (NGOs). Focus of resource: (i) principles/practice of prudent prescribing; (ii) antimicrobial stewardship principles/practices; (iii) guidelines/policies/pathways for syndrome management of infections; (iv) infection prevention/control; (v) implementation/behaviour change; (vi) evaluation/measurement; (vii) evidence gathering. Target audience: (i) doctors; (ii) pharmacists; (iii) nurses/midwives; (iv) non-medical managers; (v) public health; (vii) laboratory; (viii) infection prevention practitioners. Relevance/applicability: LIC, low-income country; LMIC, low- and middle-income country; HMIC, high- and middle-income countries; HIC, high-income country. Setting of resource: (1) pre-service (university, higher education institution); (2) service: (i) hospital; (ii) outpatient clinic; (iii) community/general practice; (iv) long-term care facility/nursing home; (v) hospital and ambulatory; (vii) other.

Our search strategy identified a total of 1191 citations, of which 6 were selected for final review. Resource identification and selection processes are detailed in Figure 1. The final list of resources varied in the platform utilized to communicate information as well as in the approach. Four resources included diverse instructional web-based implementation toolkits that focused on penicillin-allergy evaluation and subsequent management. Another resource featured a mobile app that provided a point-of-care risk assessment calculator for clinicians to use at the bedside when evaluating patients with reported penicillin-allergy. The last resource included a video supplement that highlighted useful materials for clinicians interested in performing penicillin-allergy testing in their practice. Characteristics of each resource are described in Figure 2.

### Penicillin-allergy delabelling toolkits

Four penicillin-allergy delabelling toolkits created by reputable organizations within three countries were included. Together, the toolkits provided a wide-range of adaptable clinician and patient materials, from drug-challenge compounding recipes to patient consent forms and clinician-to-clinician communiqués. Most content was directed at GP with limited penicillin-allergy assessment and delabelling experience, although some resources did require a higher baseline understanding of penicillin-allergy. Toolkit offerings are compared in Table 1 and specified below in alphabetical order.

**Table 1.** Comparison of characteristics between penicillin-allergy toolkits

Feature of penicillin-allergy toolkit	AAAAI	BC PACE	AHRQ	SAPG
Components of a penicillin-allergy history		✓	✓	
Penicillin-allergy history risk stratification		✓	✓	✓
Management of high-risk populations (pregnant, paediatric)		✓		
Drug challenge protocol		✓		✓
Drug challenge consent form				✓
Penicillin skin-testing protocol		✓		
Drug challenge and penicillin skin-testing compounding recipes		✓		
Allergy wallet card		✓		✓
Management of adverse drug reactions		✓		✓
β-Lactam cross-reactivity		✓		
Clinician-to-clinician communication letter for allergy test results				✓
Clinician-to-patient communication letter for allergy test results		✓		✓
Hyperlinks to reputable penicillin-allergy papers; podcasts	✓			
Clinician educational videos^a^	✓		✓	
Clinician educational FAQ				✓
Patient educational infographics and posters	✓			
Written patient educational materials	✓			✓
Social media graphics	✓			
Government/promotional advocacy documents	✓			

a Contents of educational video not included in table.

#### Agency for Healthcare Research and Quality (AHRQ): penicillin-allergies and other side effects of antibiotic use

The AHRQ had several antibiotic stewardship toolkits on their website, one of which was titled ‘Penicillin Allergies and Other Side Effects of Antibiotic Use’.^18^ This webpage included a video and documents about the diagnostic approach to patients with a penicillin-allergy label, how to conduct a detailed medication allergy history, and cross-reactivity between β-lactam antibiotics. There was minimal information specifically about penicillin-allergy delabelling but the document that served as a guide to interview patients with a penicillin-allergy label and assess risk of future hypersensitivity may be helpful to identify patients that may qualify for delabelling. Notably, this document did not include recent data assessing hypersensitivity risk as the PEN-FAST calculator did (see below).

#### AAAAI: penicillin Allergy Center

The Penicillin Allergy Center from the AAAAI provided a centralized clinician repository for resources related to penicillin-allergy.^19^ Two short videos (<10 min each) reviewed updates in adult and paediatric penicillin-allergy. Also included were references like readings and podcasts, links for promoting penicillin-allergy awareness (e.g. infographics, social media posts) and advocacy resources. Clinicians may find the practice resources, including coding information, to be particularly helpful.

Several strategies for delabelling were included in the videos. First, telemedicine evaluations may be useful for low-risk patients who do not need an in-person visit (i.e. family history of penicillin-allergy) or for those with unknown risk to determine if an in-person evaluation is needed.^20,21^ Second, persons in a hospital for another reason may benefit from an e-consult with a penicillin-allergy specialist.^22,23^ In this strategy, any evaluation or delabelling could be performed in the hospital under medical supervision, even without an on-site specialist. Both of these interventions have been associated with decreased time to evaluation, decreased patient wait time, and increased patient satisfaction. Next, data from a paediatric population suggest that the vast majority of patients tolerate penicillins, and skipping the skin-prick test and 10 day challenges are a viable strategy for conducting successful single-day oral challenges, leading to more time-efficient delabelling in this population.^24,25^ Finally, educational initiatives for improving delabelling in populations with high antibiotic exposure were reviewed, including education to surgical personnel resulting in an increase in peri-operative cephalosporins that were historically avoided due to allergy cross-reactivity, and education to paediatricians related to recognizing peri-infectious syndromes that present similarly to antibiotic allergies.^26^

Although this helpful repository was up to date (information updated within the past year), it required additional clicks to gather information. As such, this reference may be better suited for clinicians who are already familiar with penicillin-allergy processes and are simply looking for a specific article or resource to share or for those who need a place to start and have time to explore.

#### British Columbia (BC) Provincial Antimicrobial Stewardship Clinical Expert (PACE) group: β-lactam allergy delabelling guideline and toolkit

The BC PACE group is comprised of members from the Ministry of Health, local infection control network, and local antimicrobial stewardship programmes. This group serves to assist in antimicrobial stewardship endeavours at acute care and long-term care facilities throughout the province in Canada. In 2021, the group created and endorsed the ‘Beta-lactam Allergy Delabeling Guideline and Toolkit’.^27^ The goal was to provide a concise document that can be utilized for large-scale intervention and behaviour changes for facilities to implement standardized programmes based on the resources available. Because the document was downloadable and searchable, even a clinician with limited time could find specific actionable interventions that can be implemented in their practice. The toolkit served as a .pdf document organized into general background information on allergies, specifically penicillin, and how to interview, risk stratify and directly intervene to delabel. There was a specific section dedicated to cross-reactivity amongst β-lactams, and when to otherwise consider referral to an allergy specialist. A unique aspect of this reference was that it was targeted to the GP, namely physician or pharmacist, in order to help guide day-to-day practice on best approaches for the patient that presents with a β-lactam allergy and can be applied to individual facilities. Most importantly, and what made this reference a toolkit, were the tables and supplementary materials provided at the end of the document. Many of these were meant to be used and adapted by others to best fit practices and resources available within individual institutions. This would also include underserved areas in resource-limited settings. Much of the information was geared toward assessment, so even a resource-limited setting would be able to take away actionable interventions. The toolkit was supported by primary data, and many of the supplementary materials and protocols were adapted from published literature. It was noted before the supplementary material that this toolkit was created in 2021 and an audit trail can be followed for any updates.

#### Scottish Antimicrobial Prescribing Group (SAPG): penicillin-allergy delabelling

The SAPG was established by the government in 2008 to promote effective and safe antimicrobial use across inpatient and outpatient clinical settings by partnering with clinical healthcare providers. One of the quality improvement tools developed by SAPG was specific to penicillin-allergy delabelling.^28^ While some documents were comprehensive toward all penicillin-allergy intervention strategies, this toolkit focused on direct challenge of oral penicillins in low-risk patients. Some of the clinician resources included protocols for implementing penicillin-allergy delabelling within acute care hospitals as well as patient risk assessment and allergic-reaction algorithms. Several practical resources for clinicians were provided as well, including sample letters detailing positive or negative reactions to penicillin testing to general practitioners (GPs) as well as sample allergy cards to give to patients. A comprehensive ‘frequently asked questions’ (FAQ) document was provided, which detailed some of the most common pre-test clinician questions, pre-test patient penicillin-allergy scenarios, and intra-test as well as post-test clinician questions. This toolkit also provided several resources for patients including pre-test information, a sample patient permission form to be signed prior to penicillin challenge, and patient leaflets for both a positive and a negative penicillin-allergy result with additional instructions including next steps for both the prescriber and the patient. Because of the nature of the resources, most did not contain references (except patient risk/consent), but overall recommendations were consistent with known published data within penicillin-allergy assessment and intervention strategies. The information provided was practical in nature, consisting of a number of documents that may easily be downloaded and modified. These resources would be a good starting point for those wishing to initiate direct oral penicillin challenges in low-risk patients within an acute care population. There is potential for many of these same principles to be applied to outpatient settings through appropriate risk stratification as outlined within the toolkit as long as appropriate acute management of reactions could be ensured similar to an inpatient setting. One exclusion criterion for the algorithm is ‘currently requiring antibiotic treatment for a severe infection’. This criterion may be overly conservative and may not apply to specific institutions wishing to proceed with delabelling, as long as the patient is haemodynamically stable and a low-risk penicillin-allergy can be verified. This toolkit was published in October 2021 and is due to be re-evaluated formally in October 2024. The SAPG encouraged ongoing feedback, which may be provided via e-mailing them directly.

### Penicillin-allergy risk assessment calculator

Limited clinical decision support tools to assist with penicillin-allergy delabelling were discovered in the grey literature. One readily and freely accessible mobile app was included in the final review.

#### ‘Calculate by QxMD’ electronic app: PEN-FAST—penicillin-allergy risk tool

The PEN-FAST penicillin-allergy risk tool calculates the likelihood of a patient having a positive penicillin-allergy skin test and enables point-of-care risk assessment of patient-reported penicillin allergies.^29^ The PEN-FAST calculator was created using data from 622 patients reporting a penicillin-allergy who prospectively underwent allergy testing via skin prick, intradermal or patch testing and/or oral challenge.^30^ The clinical variables associated with positive penicillin-allergy test results were distilled into a mnemonic, PEN-FAST. It requires three clinical criteria: time from last hypersensitivity from penicillin, the hypersensitivity reaction symptoms, and if there was any medical treatment required for the reaction. Based on the details of the clinical criteria, the PEN-FAST risk tool will determine if the patient is very low risk of a positive penicillin-allergy test (<1%), low risk of a positive penicillin-allergy test (5%), moderate risk of a positive penicillin-allergy test (20%) or high risk of a positive penicillin-allergy test (50%). The low-risk group represented 74% of the cohort, of which only 17 of 460 patients (3.7%) had a positive test result. The negative predictive value was 96.3%. External validation of the PEN-FAST decision aid remained clinically relevant in a retrospective cohort of 945 patients from three centres in Sydney and Perth, Australia and Nashville, TN, USA. This study supports identifying patients with low risk for true penicillin-allergy and safe use of this drug class, perhaps through an observed oral challenge in a primary care setting.

### Supplemental instructional video

Educational videos were frequently encountered when screening grey-literature citations; however, few aimed to provide actionable delabelling strategies. One instructional video featuring standardized approaches to assessing and managing penicillin-allergy into clinical practices was identified.

#### Journal of the American Medical Association (JAMA) network: penicillin-allergy—evaluation and testing (YouTube video)

This reference was a combined live and animated video on YouTube and published by JAMA Network, a YouTube channel with >180 000 subscribers.^31^ The video itself was just under 20 min long and had over 73 000 views to date. The video highlighted the important, actionable items from the JAMA article, ‘Evaluation and Management of Penicillin Allergy: A Review’ published in 2019, along with the supplemental toolkits that accompany the article.^32^

This video was unique and effective because it employed animated graphics and pictures, which help viewers to visualize and better understand penicillin-allergy testing techniques, anaphylaxis kits and allergic skin reactions. Additionally, use of the toolkits was demonstrated within the video. This video also utilized segmented breaks to aid the viewer in isolating the topic of interest, such as the level of risk of allergic reaction (low, moderate, high), thereby enhancing the time-saving usefulness for busy clinicians. This video provided valuable advice on performing the oral amoxicillin challenge for low-risk, ambulatory adults.

Unfortunately, this video was only applicable to outpatient, non-pregnant adults in clinics and provider offices that elect to perform penicillin-allergy testing. Further, while the video showed examples of allergic reactions from intradermal and skin-prick testing, the examples were only on white skin, so determining the extent of allergic reactions on darker skin tones might be more challenging. Lastly, while the risk of anaphylaxis is extremely rare with penicillin skin testing and the oral amoxicillin challenge, it is prudent to have an anaphylaxis kit nearby as a precaution, which may not be possible in some resource-limited settings.

## Discussion

This scoping review produced a bundle of penicillin-allergy delabelling resources that offer freely accessible and unique evidence-based strategies for clinicians to integrate into their daily practices around the world. To our knowledge, this is the first exploratory review that mapped grey-literature materials on penicillin-allergy delabelling for use by non-allergy clinicians, including those practising in resource-limited settings. The various delabelling methods presented in this review allow programmes to target specific pressure points based upon their facility practice needs and available resources: penicillin-allergy toolkits provide a collection of adaptable resources to assist clinicians with implementing practice changes; use of mobile app calculators for point-of-care penicillin-allergy assessments promotes direct allergy delabelling in select low-risk patient populations; lastly, instructional videos engage clinicians on strategic delabelling approaches and effective initiatives. Application of one or more of these strategies into routine clinician practices across the globe has the potential to neutralize the negative effects associated with the penicillin-allergy label.

Four penicillin-allergy toolkits were identified through our search method. It is particularly interesting to assess how each organization defined its toolkit and which materials were prioritized. No one toolkit provided guidance for all delabelling practices offered, as illustrated by Table 1. Modifiable documents allow clinicians to individualize practices based on facility culture and can assist with the upfront work required with new programme or initiative development. The target audiences for some documents contained within these toolkits extend beyond clinicians and patients to also include members of Congress, social media followers, and local news subscribers. It should be noted, however, that the effectiveness of each toolkit, in its entirety, on clinical or financial outcomes has not been evaluated.

The PEN-FAST risk assessment calculator and the supplemental instructional video add diversity in strategic approach to the resource list. The calculator can easily and reliably detect low-risk penicillin-allergic patients. Identification of patients through this pathway may facilitate direct delabelling in some, or drug challenges in others. The instructional video highlights a recently published review of penicillin-allergy by content experts.^32^ Although the review article is not publicly available to non-subscribers, the journal provides a free video that summarizes the article and includes images of the toolkits to those that are unable to access the full text. Visual learners may also benefit from the animated storytelling used throughout the lesson.

There is currently no reference standard for how to approach and appropriately intervene on a patient with a listed, subjective allergy to penicillin. Without formal training in allergy and immunology, the GP is often unable to incorporate clarification as a high-priority intervention. This review constructed a foundational layer of delabelling methods for clinicians or programmes to consider but identifying actionable and literature-supported strategies was difficult to tease out of the grey literature. Of the nearly 1200 citations found in our search, only 6 were considered to have answered the research question, meaning that a relatively limited number of references were identified that could help resource-limited settings implement delabelling programmes. National health protection agencies and infectious diseases organizations claim to support penicillin-allergy delabelling programmes but stop short of providing strategies that can accomplish such goals in these settings. Endorsement of available resources by premier organizations would be instrumental in circulating impactful practices that promote penicillin-allergy evaluation and delabelling programmes. Expansion of future resource materials that would greatly contribute to existing databases may include toolkits and patient education in non-English languages, guidance on electronic health solutions to penicillin-allergy delabelling that could affect patients by the masses, and support of international drug allergy registries to help characterize drug allergy and associated needs in other areas of the world.

Several limitations were identified in this scoping review. First, robust epidemiological and outcome data for most countries were not available. Statistics and outcomes related to allergist shortages and performance of resources (such as the PEN-FAST calculator) were extrapolated from published findings predominantly out of the USA and Australia. Second, our database searches were limited to resources published in the English language from select websites, potentially excluding relevant articles published outside of these parameters. Third, although the authors attempted to avoid Google-based search engines, some organizational search fields utilized this web browser, which may have generated irreproducible and biased search results. Last, given the inherent nature of scoping reviews, our search strategy required multiple approaches to identifying sources, including manual screening of citation titles and topics.

### Conclusions

This scoping review broadly characterizes penicillin-allergy delabelling strategies published in the grey literature for application by practitioners in resource-limited settings. Using a focused search, we identified six resources that openly promote evidence-based and actionable delabelling solutions to penicillin-allergy. When used collectively, these resources provide diverse opportunities to standardize and improve the management of penicillin-allergy and facilitate knowledge and translation of ideas into clinical care at individual facilities. Additional support and consensus from health protection agencies is critical to augment ongoing delabelling efforts and improve patient and health-related outcomes on a global level.
